# Larval Mid-Gut Responses to Sub-Lethal Dose of Cry Toxin in Lepidopteran Pest *Achaea janata*

**DOI:** 10.3389/fphys.2017.00662

**Published:** 2017-09-05

**Authors:** Vinod K. Chauhan, Narender K. Dhania, R. K. Chaitanya, Balasubramanian Senthilkumaran, Aparna Dutta-Gupta

**Affiliations:** ^1^Department of Animal Biology, School of Life Sciences, University of Hyderabad Hyderabad, India; ^2^Centre for Animal Sciences, School of Basic and Applied Sciences, Central University of Punjab Bathinda, India

**Keywords:** cry toxin, lepidopteran pest, gut regeneration, mid-gut stem cells, arylphorin

## Abstract

The lack of homogeneity in field application of *Bacillus thuringiensis* formulation often results in ingestion of sub-lethal doses of the biopesticide by a fraction of pest population and there by promotes the toxin tolerance and resistance in long term. Gut regeneration seems to be one of the possible mechanism by which this is accomplished. However, the existing information is primarily derived from *in vitro* studies using mid-gut cell cultures. Present study illustrates cellular and molecular changes in mid-gut epithelium of a *Bt*-susceptible polyphagous insect pest castor semilooper, *Achaea janata* in response to a Cry toxin formulation. The present report showed that prolonged exposure to sub-lethal doses of Cry toxin formulation has deleterious effect on larval growth and development. Histological analysis of mid-gut tissue exhibits epithelial cell degeneration, which is due to necrotic form of cell death followed by regeneration through enhanced proliferation of mid-gut stem cells. Cell death is demonstrated by confocal microscopy, flow-cytometry, and DNA fragmentation analysis. Cell proliferation in control vs. toxin-exposed larvae is evaluated by bromodeoxyuridine (BrdU) labeling and toluidine blue staining. Intriguingly, *in situ* mRNA analysis detected the presence of arylphorin transcripts in larval mid-gut epithelial cells. Quantitative PCR analysis further demonstrates altered expression of arylphorin gene in toxin-exposed larvae when compared with the control. The coincidence of enhanced mid-gut cell proliferation coincides with the elevated arylphorin expression upon Cry intoxication suggests that it might play a role in the regeneration of mid-gut epithelial cells.

## Introduction

*Bacillus thuringiensis* (*Bt*) strains synthesize different toxins including crystal (Cry), cytolytic (Cyt), and vegetative insecticidal proteins (Vip). While Cry and Cyt toxins are synthesized during sporulation (Hofte and Whiteley, 1989; Bravo et al., 2007), Vip toxins are produced during the vegetative growth phase (Estruch et al., 1996). Lepidopteran larvae experimentally used to demonstrate the mode of action of Cry toxin, showed sequential event of toxin solubilization, its activation induced by alkaline pH of the gut, proteolytic cleavage, binding of toxin with different receptor molecules, oligomerization, and membrane insertion leading to pore formation and epithelial cell damage (Bravo et al., 2007). Further, *in vitro* studies suggested that the Cry toxin-induced cell death is primarily of necrotic type (Zhang et al., 2016).

*Bt* and its insecticidal toxins have been predominantly used for the control of maize and cotton pests (Bravo et al., 2011; Hellmich and Hellmich, 2012). The development of insect resistance to *Bt* is a major concern for its long-term effectiveness. Extensive studies revealed, alteration in mid-gut binding sites leading to reduced interaction of *Bt* toxins to their receptor, as a major mechanism for the development of resistance (Pigott and Ellar, 2007). Since many Cry toxins share common binding sites, the probability of cross-resistance was often found to be high (Ferre and Van Rie, 2002; Tabashnik et al., 2013). In agreement with these reports, insects such as diamond black moth, *Plutella xylostella*; Indian meal moth, *Plodia interpunctella*, and cabbage looper, *Trichoplusia ni* have evolved resistance to commercial *Bt* sprays in the open field and greenhouses (Ferre et al., 1991; Oppert et al., 1997; Janmaat and Myers, 2003). Recently, field-evolved pest resistance to *Bt* corn (maize stem borer, *Busseola fusca*; western corn rootworm, *Diabrotica virgifera virgifera*; and fall armyworm, *Spodoptera frugiperda*), *Bt* cotton (cotton bollworm, *Helicoverpa armigera*, and pink bollworm, *Pectinophora gossypiella*), and *Bt* maize (*Spodoptera frugiperda*) have also been reported (Storer et al., 2010; Dhurua and Gujar, 2011; Kruger et al., 2011; Zhang et al., 2011; Jakka et al., 2015; Pereira et al., 2015).

Additional physiological responses from larvae alike non-receptor mediated resistance mechanisms could interfere with steps common to all Cry toxins vis-à-vis cross-resistance to a wide range of *Bt* toxins. One such process is the enhanced mid-gut regeneration response observed in the resistant larvae. Lepidopteran larvae have been shown to recover from exposure to sub-lethal Cry toxin doses (Dulmage and Martinez, 1973) by mid-gut epithelial regeneration (Spies and Spence, 1985). Further few microscopic studies showed enhanced mid-gut healing response to facilitate Cry toxin resistance in the tobacco budworm, *Heliothis virescens* (Forcada et al., 1999; Martinez-Ramirez et al., 1999). However, there is a dearth of information on the molecular aspects of mid-gut epithelial response to Cry toxin. In addition, most of the information on the regenerative process is derived from *in vitro* studies using primary mid-gut cell cultures (Loeb et al., 2001; Blackburn et al., 2004; Hakim et al., 2010) mainly due to the lack of mid-gut specific biomarkers to allow quantitative detection of lepidopteran mid-gut cells, their proliferation as well as differentiation. The *in vitro* data circumvents specific cell/tissue interactions within the insect system. One of the candidate molecules presumed to be involved in the mid-gut response to *Bt* toxins is arylphorin, which belongs to the hexamerin protein family of insects. Arylphorin was shown to stimulate stem-cell proliferation *in vitro* as well as upon feeding with diet, where it promoted the growth of gut suggesting its mitogenic effect on gut cells (Blackburn et al., 2004; Hakim et al., 2007).

Foliar spray of *Bt* formulation often results in the loss of toxicity from degradation by UV light, wash-off by rain, drying, temperature, and soil acidity as well as its chemistry (Pinnock et al., 1975; Leong et al., 1980; Beckwith and Stelzer, 1987; vanFrankenhuyzen and Nystrom, 1989). Under afore mentioned conditions, there is always a possibility of a population of larva to get exposed to sub-lethal doses of toxin which might exhibit a variable effect and escape mortality. In line with these observations the present study is aimed to evaluate the effect of sub-lethal doses of *Bt* toxin on mid-gut of the larva of *Achaea janata*, an economically important polyphagous pest prevalent in the Indian subcontinent. *In vivo* analysis document the necrotic type of cell death associated with regeneration of mid-gut epithelial cells during Cry intoxication. Additionally, the presence of arylphorin transcript in larval mid-gut cells and its differential expression during Cry intoxication is also demonstrated.

## Materials and methods

### Insect rearing and maintenance

For the present study, larvae of *A. janata* Linn. (Noctuidae: Lepidoptera) were collected from the fields of Indian Institute of Oil Seeds Research, Hyderabad with no prior exposure to any synthetic or *Bt* pesticides, reared in the laboratory conditions (Budatha et al., 2007). They were maintained for three generations as to obtain homogenous population (in a growth chamber). Larvae were fed with sterile castor leaves and maintained at 27 ± 2°, 14:10 h (light: dark) photoperiod and relative humidity of 60–70% till pupation. Pupae were transferred to moist sand container for adult emergence. Adults were transferred to egg laying chamber and were supplemented with 10% honey containing Vitamin E. Adults laid their eggs on the leaves and the neonate larvae hatched were used for continuous culture as well as experiments.

### Administration of DOR *Bt*-1 formulation

DOR *Bt*-1 formulation was obtained from Indian Institute of Oil Seeds Research which exhibited high toxicity against *A. janata* larvae and various other lepidopteran pests. The PCR analysis of DOR *Bt*-1 isolate revealed presence of *cry1* (*cry1Aa, cry1Ab*, and *cry1Ac*) and *cry2* (*cry2Aa* and *cry2Ab*) genes, which provide broad-spectrum potential against lepidopteran, and dipteran insects (Reddy et al., 2012). The reported LC_50_ value for the DOR *Bt*-1 formulation was 247.52 μg/ml for seven-day-old or 3rd instar larvae (Vimala Devi and Sudhakar, 2006). One tenth of LC_50_ value was used as a sub-lethal dose (i.e., 24.75 μg/ml of water) for the present study. Toxin coated castor leaves were fed to the larvae. Triplicates were maintained, for each treatment 150 larvae were used. They were divided into two sets of experiments, wherein one batch was continuously exposed to sub-lethal dosage of toxin (0.170 μg/cm^2^) coated castor leaf discs (145 cm^2^) till 72 h, and a batch of 30 larvae were intermittently transferred to uncoated leaf discs after every 24 h period of toxin exposure for the recovery. Control larvae were maintained on water coated leaf discs. Furthermore, morphological changes occurring during the development and mortality were also recorded in toxin-exposed as well as recovered insects.

### Tissue isolation

Third instar larvae (a batch of 100) exposed to toxin coated leaves were collected at every 12 h interval till 72 h. These were briefly narcotized on ice and dissected in insect Ringer solution (130 mM NaCl, 0.5 mM KCl, and 0.1 mM CaCl_2_). An incision was made in the abdominal region and extended through the length of the larvae. The gut content was removed and the mid-gut region was specifically isolated. After removal of the gut, the yellowish-white perivisceral fat body was collected. The isolated tissues were used for histology, *in-situ* hybridization, genomic DNA and total RNA isolation.

### Mid-gut cell death analysis

Cell death/viability of mid-gut cells was primarily assessed using AnnexinV and Propidium Iodide (PI) staining (Vermes et al., 2000). For this experiment mid-gut was dissected out from both control and toxin exposed (24 h) larvae and maintained in Grace insect cell culture media (Invitrogen Corporation, USA) and incubated with AnnexinV and Propidium Iodide staining solutions (BD Biosciences, UK) and visualized under confocal microscope (LSM 880, Carl Zeiss, Jena). Furthermore, gut cells were strained through 70 μm mesh size MACS® smart strainer (Miltenyi Biotec, Singapore) using phosphate buffer saline (PBS) solution (pH 7.4). The cell reservoir was collected in the funnel and centrifuged at 1,500 rpm for 5 min at 4°C. The cell suspension was collected and immediately processed for FACS (fluorescence-activated cell sorting) analysis. An analysis was carried out using Annexin V-FITC Apoptosis Detection Kit according to the manufacturer's instructions (BD Biosciences, UK). In brief, AnnexinV and Propidium Iodide staining solutions were added to the sieved cell suspension and incubated at room temperature in the dark for 15 min. This was followed by addition of binding buffer and then analyzed using a FACS LSR Fortessa^TM^ flow cytometer (BD Biosciences, UK) and BD FACS Diva Software 6.0. For each sample, 10,000 events were acquired. The percentage of live cells vs. cells undergoing death was plotted.

To substantiate that cell death is due to necrosis but not because of apoptosis in larval mid-gut, DNA fragmentation assay was carried out following the protocol of Kasibhatla et al. (2006). Genomic DNA from the mid-gut was isolated using a standard protocol (Lagisz et al., 2010), with minor modifications. The tissue (100 mg) was homogenized using 500 μl of lysis buffer (100 mM Tris-HCl (pH8.0), 50 mM EDTA, 1.5 mM NaCl, 2% CTAB, and 0.1% 2-mercaptoethanol). DNA pellet thus obtained was solubilized in Tris-EDTA buffer (pH 8.0) and analyzed by 0.8% agarose gel electrophoresis.

### Histological analysis of mid-gut

The isolated mid-guts were fixed overnight in Bouin's fixative (saturated picric acid: formaldehyde: glacial acetic acid; 15:5:1), processed through a series of increasing ethanol concentrations, cleared in xylene and embedded in molten paraplast (Sigma-Aldrich, USA). The tissues were sectioned (~5 μm) using a rotatory microtome (Leica Microsystems, Germany) and stained with haematoxylin-eosin or toluidine blue (Sousa et al., 2010), visualized using Olympus IX-81 microscope (Olympus Corporation, Japan), and photographed.

### Mid-gut cell proliferation assay

Streptavidin-biotin based BrdU staining (Invitrogen Corporation, USA) was used for monitoring the cell proliferation in the mid-gut during toxin-induced damage following manufacturer's protocol. Larvae were injected with BrdU labeling reagent (10 μl/g body weight) 4 h prior to isolation of toxin exposed larval mid-gut using a Hamilton syringe. Dissected mid-guts were fixed and processed as mentioned in the previous Section Histological Analysis of Mid-Gut, tissue sections were spread on poly-L-lysine coated slides (Sigma-Aldrich, USA) and incubated overnight at 37°C following which deparaffinization was carried out using xylene and passed through a series of decreasing ethanol concentrations. Peroxidase quenching was performed using 30% H_2_O_2_ and absolute methanol (1:9). Denaturation and blocking steps were carried out using the kit based reagent prior to antibody incubation. Biotinylated mouse anti-BrdU was overlaid and the slides were incubated at room temperature (RT). After incubation, streptavidin peroxidase (Invitrogen Corporation, USA) was added to detect the incorporation. Color development was monitored after the addition of secondary antibody mixture provided by the manufacturer. Slides were counterstained and mounted using histomount™ (Invitrogen Corporation, USA) and visualized under Olympus IX-81 microscope.

### *In situ* localization of arylphorin mRNA

For the preparation of arylphorin cRNA probe, a 160 bp fragment of *A. janata* arylphorin gene was positioned as forward locked in pGEM-T Easy Vector (Promega Corporation, USA). The vector was linearized with EcoRI and NdeI restriction enzymes (New England Biolabs®, UK). Sp6 promoter was used for the synthesis of antisense probe (positive), while T7 promoter was used for the synthesis of sense probe (negative). Further, RNA probe synthesis was carried out using DIG RNA labeling kit (Roche Diagnostics, Germany). Larval mid-gut and fat body were dissected out and fixed in 4% paraformaldehyde (PFA) (Sigma-Aldrich, USA) in 0.1 M PBS at 4°C overnight. Post-fixation, tissues were embedded in OCT compound medium (Leica Microsystems, Germany). Tissue cross sections were obtained using cryostat microtome (Leica CM1850, Leica Microsystems, Germany) and mounted on poly-L-lysine coated slides. The OCT compound was washed using PBST buffer and the sections were permeabilized using proteinase K (1 μg/ml). Once again 4% PFA was used for further tissue fixation and sections were incubated with 200 μl hybridization buffer (50% formamide, 5X SSC, 5 mM EDTA, 0.1% CHAPS, 1% SDS, 50 μg/ml yeast tRNA, 50 μg/ml heparin) for 1 h at 50°C. The digoxigenin labeled cRNA probe was mixed with 200 μl of hybridization buffer, heat denatured at 80°C for 5 min, draped and covered with parafilm and incubated at 50°C in a sterile incubator overnight. Post-hybridization, the slides were washed four times at 50°C (5 min each) with a wash buffer (SSC, 50% formamide, 0.1% Tween 20) in a series of decreasing SSC concentration. The slides were then processed with solution A (0.5 M NaCl, 5 mM EDTA, 10 mM Tris-HCl, 0.1% Tween 20, pH 8.0) and incubated at RT for 5 min followed by incubation with 20 μg/ml of RNase A for 20 min at RT. Later, the slides were again washed with solution A twice (5 min each) at RT.

For the antibody reaction, the slides were initially washed with maleic acid buffer (100 mM maleic acid, 150 mM NaCl, 0.1% Tween 20, pH 7.5) for 5 min at RT followed by blocking with 10% BSA for 1.5 h at RT. Anti-digoxigenin-ALP (Roche Diagnostics, Germany) was used for the detection of digoxigenin probe (1:2,000) and incubated overnight at 4°C. These slides were then washed with DIG wash buffer (Roche Diagnostics, Germany) for 4 times (15 min each). Incubation with 100 μl BCIP-NBT solution (G-Bioscience, USA) for 10 h at RT was carried out to develop the color. After color development, the slides were overlaid with detection buffer (Roche Diagnostics, Germany), counterstained with nuclear red (Vector Laboratories Inc., USA), and incubated in TE buffer (Ambion, Life Technologies, USA) for 5 min. After washing with PBST buffer, the slides were processed with increasing series of ethanol, mounted using DPX mountant, and visualized under Olympus IX-81 microscope.

### Arylphorin mRNA expression analysis

Total RNA was isolated from the tissue (100 mg) using TRIzol method® (Ambion, Life Technologies, USA). The quantification of the RNA was carried out using NanoDrop spectrophotometer (ND-2000; NanoDrop Technologies, USA). Quality and integrity of RNA were assessed by formaldehyde denaturing gel electrophoresis. cDNA was synthesized using Revert Aid First Stand cDNA synthesis kit (Thermo Scientific, USA). For quantitative PCR analysis, a custom-made TaqMan gene expression assay was performed (Applied Biosystems, USA). Arylphorin primers of amplicon size 160 bp (Fwd: 5′-GTT AGA GAA GGG CCA GGT GTC TCA TG-3′; Rev: 5′- CTC TGG CTT GGT AGA CTC CTT GGT-3′) were designed from the arylphorin nucleotide sequence (KX462992). Insect rS7 gene (KF984201) was used as an internal control using primers (Fwd: 5′-ACG TGG ACG GTT CAC AAC TCA TCA-3′; Rev: 5′-TTC GCG GCC TGT TAG CTT CTT GTA-3′) which gave rise amplicon of 115 bp, to normalize the transcript expression levels. The real-time expression analysis was performed in triplicates. From the exponential phase of PCR amplification Cycle threshold (Ct) values were obtained. Arylphorin expression was normalized against the expression of rS7 rRNA to generate a ΔCt value (Ct of Arylphorin—Ct of rS7 rRNA). Comparative Ct method was used to calculate the change in the gene expression (Livak and Schmittgen, 2001).

### Statistical analysis

Presented data was calculated using standard error of the mean (SEM) (*n* = 3) which passed homogeneity and normality tests. Statistical significance was verified using the SigmaPlot v12.3 software (SystatSoftware Inc., USA). Significance between groups was tested using One-Way ANOVA followed by Student-Newman-Keuls (SNK) test for pairwise multiple comparison analysis and the *p*-value was calculated for each set of experiments.

## Results

### Cry toxin alters larval growth and development

**Figure 1:**
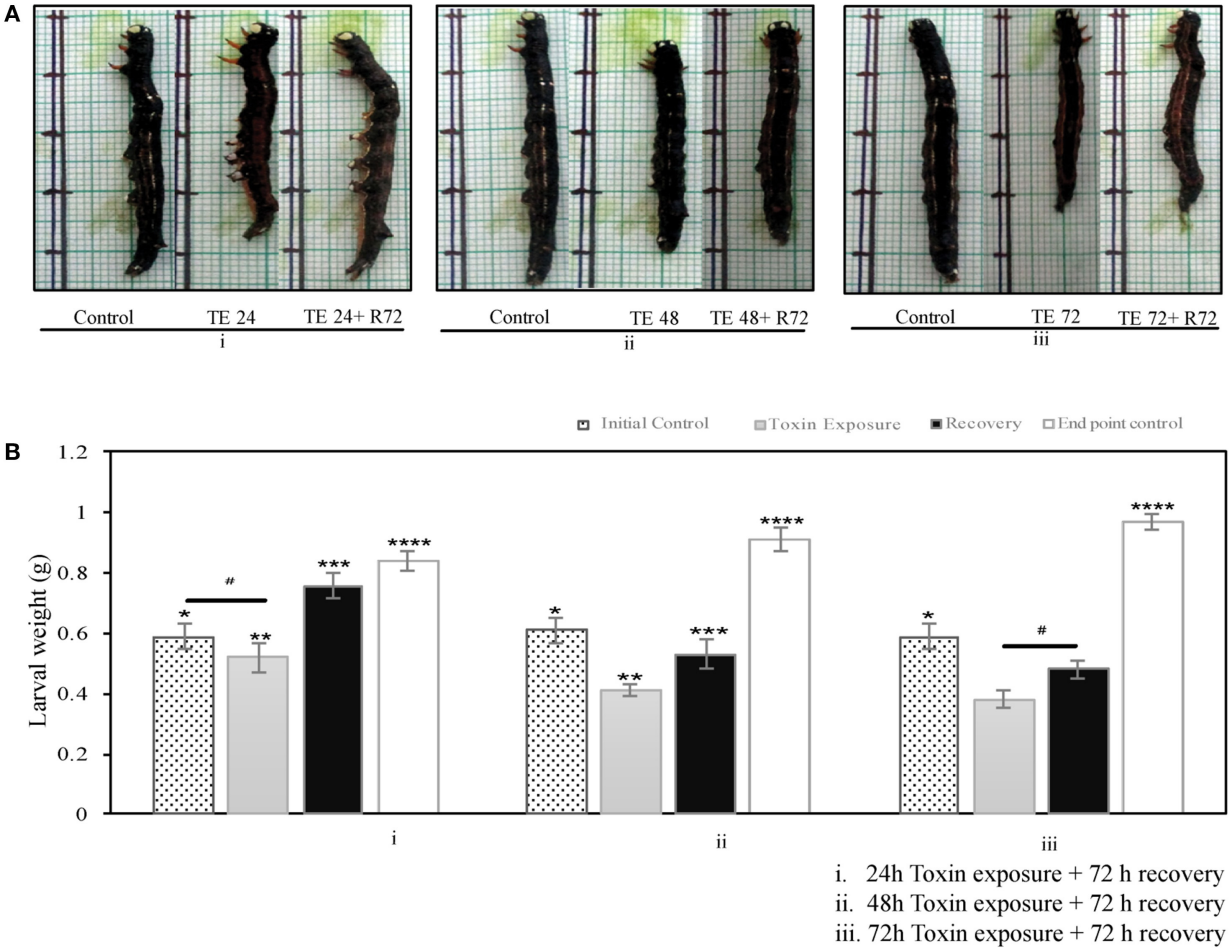
*A. janata*, third instar larvae were maintained either on Cry toxin coated or normal leaves for different time intervals- **(A)** Growth pattern seen in **(i)** 24 h, **(ii)** 48 h, and **(iii)** 72 h after Cry toxin exposure (TE), with respective controls, for recovery toxin exposed larvae were transferred to normal leaves for 72 h (R72), **(B)** Effects of Cry toxin exposure for **(i)** 24 h, **(ii)** 48 h, and **(iii)** 72 h on larval weight, and after 72 h of recovery. The values presented are the mean ± standard deviation of three independent experiments (*n* = 3), significance between groups was tested by One-Way ANOVA followed by Student-Newman-Keuls (SNK) test using SigmaPlot 12.3 software. Means marked with ^*^p < 0.05 are significant with respect to toxin exposed, recovered and age matched end point control larvae,^**^p < 0.05 indicates statistical significance with respect to recovered, and age matched end point control, ^***^p < 0.05 indicates statistical significance with respect to initial control and toxin exposed larvae, ^****^p < 0.05 indicates statistical significance with respect to the weight of initial control, toxin exposed, and recovered larvae, while # represents difference between compared group is not statistically significant.

The larvae that fed on toxin-coated leaves exhibited reduced growth in a time-dependent manner (24–72 h; Figure 1A). The 24 h toxin-exposed larvae could recover and grow when transferred to normal leaves (Figure 1B), whereas the 48 and 72 h toxin-fed groups often showed slow and poor recovery. Further, the body weight of the toxin exposed larvae was fairly low and were only 50% of that of the age matched control groups after 72 h of recovery, when the larvae were exposed to toxin for 48 and 72 h (Figure 1B).

### Mid-gut necrosis occurs in toxin-exposed larvae

**Figure 2:**
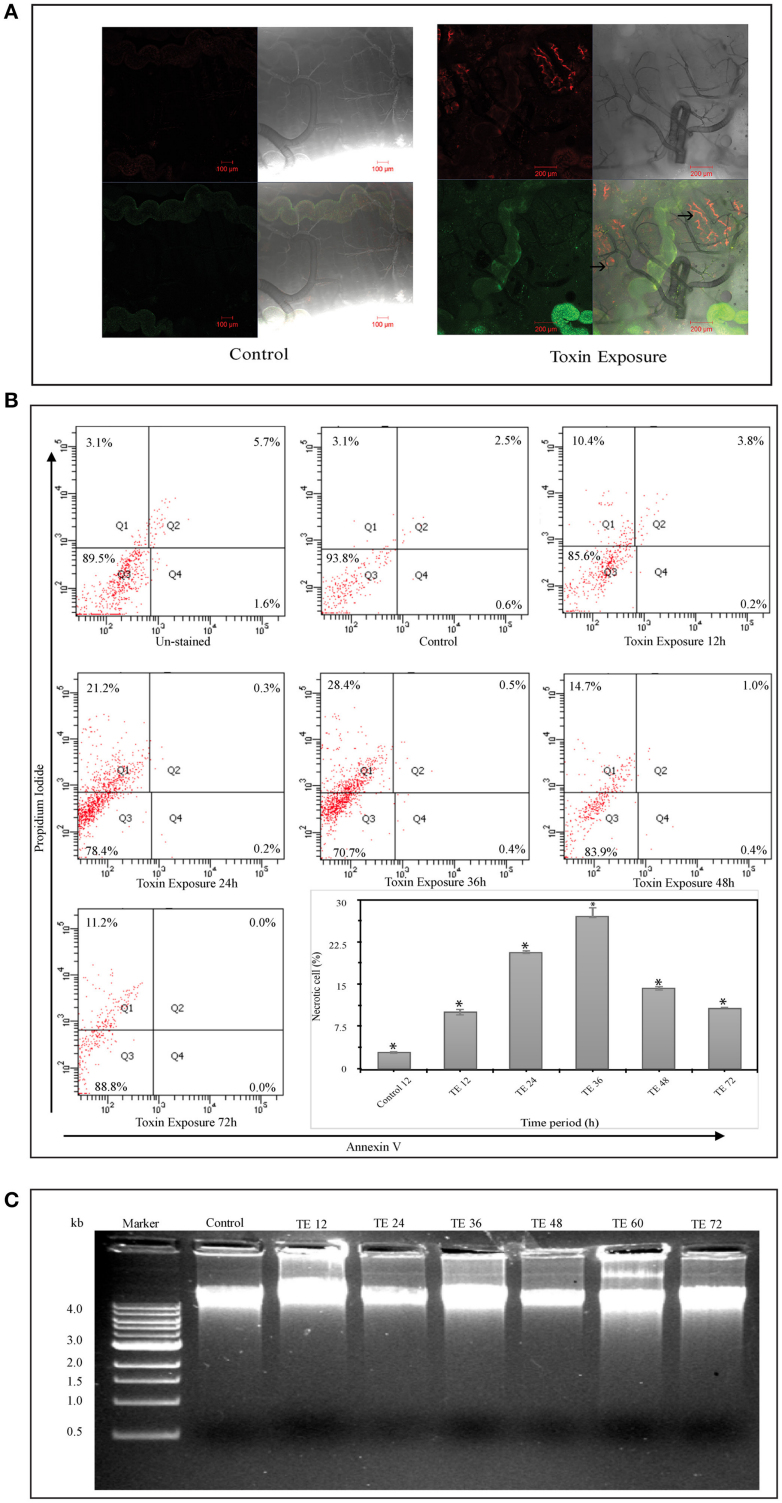
Cell death analysis using Annexin V and Propidium iodide (PI) staining and DNA fragmentation assay—**(A)** Whole mount of control and Cry toxin exposed larval mid-gut, stained with Annexin V and Propidium iodide (PI). Note the presence of PI^+^ positive cells in the mid-gut epithelium after toxin exposure (→). **(B)** FACS analysis, where scatter plot is divided into four quadrants (Q1–Q4), which give the total cell counts. Q1 represents PI^+^/Annexin V^−^ cells (necrotic), Q2 represents PI^+^/Annexin V^+^ cells (late apoptotic/necrotic), Q3 represents the viable cells and Q4 represents Annexin V^+^/PI^−^ cells (early apoptotic). Scatter plots showing the distribution of Annexin V and PI stained cells in mid-gut cell suspension prepared from control and Cry toxin exposed larvae. The cell suspension prepared from experimental larvae shows a distinct shift during 24 and 36 h of exposure into the top left quadrant (Q1). Inset shows the percentage of necrotic cells in mid-gut of toxin-exposed larvae at different points, ^*^p < 0.005 significance between experimental groups, **(C)** DNA fragmentation analysis of mid-gut shows the presence of intact band in agarose gel in both control and toxin exposed larvae after different time points.

Confocal analysis revealed the presence of PI positive cells in toxin exposed larvae but not in controls (Figure 2A). Mid-gut cell suspension prepared from toxin exposed insects showed a distinct shift into the upper left quadrant (Q1) representing necrotic type of cell death (Figure 2B). Further, the cells undergoing necrosis have increased gradually from 12 to 36 h upon toxin-exposure. However, it was noteworthy that the number of necrotic cells reduced drastically in 48 and 72 h toxin-exposed samples (Figure 2B).

The result from DNA fragmentation assay revealed that the DNA integrity was preserved in both control and toxin-exposed mid-gut samples and the absence of DNA fragmentation in all the samples rules out the possibility of apoptotic form of cell death (Figure 2C). Microscopic, FACS and DNA fragmentation analyses indicated that necrosis was the most likely reason for cell death upon Cry intoxication.

### Sub-lethal toxin exposure induces mid-gut cell proliferation

**Figure 3:**
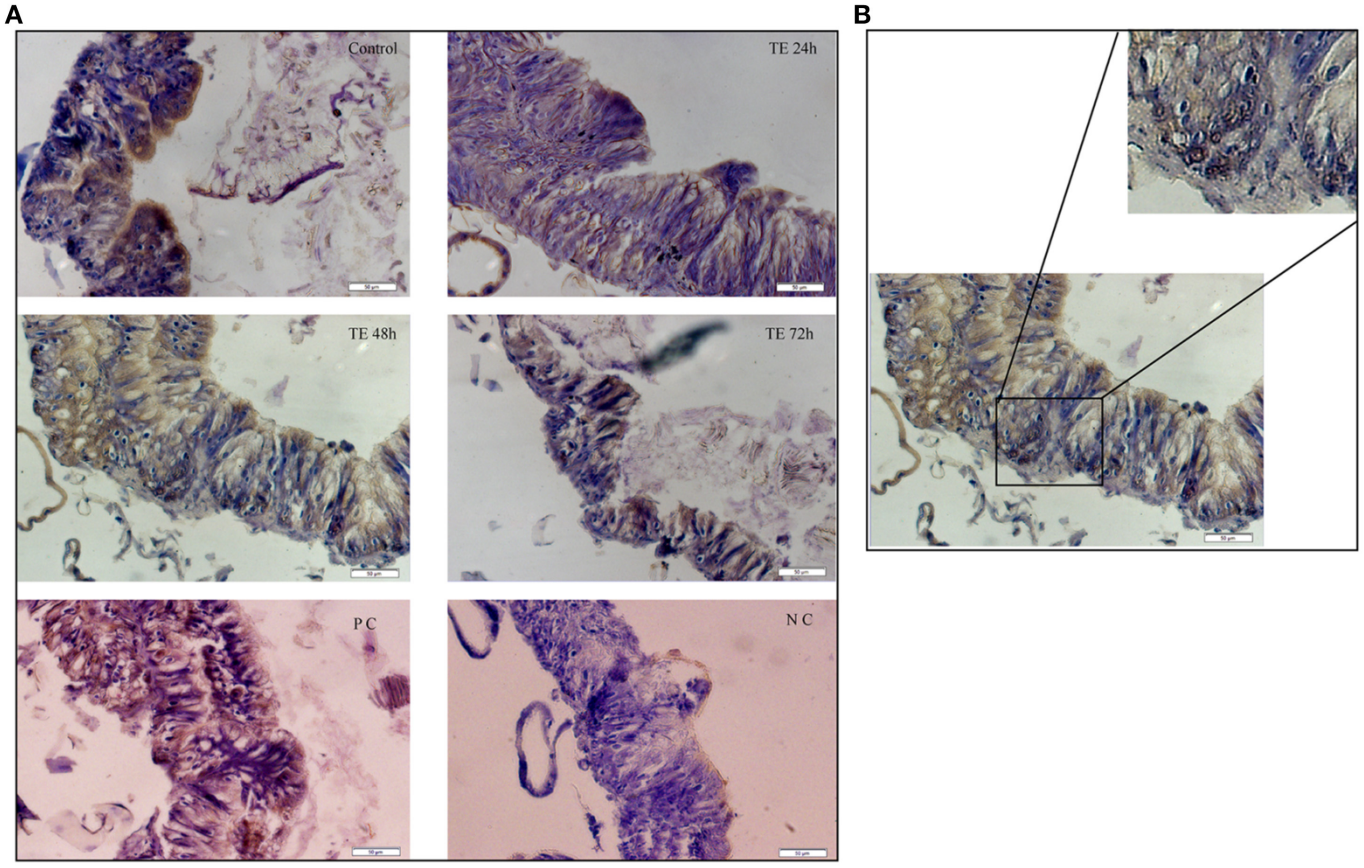
Mid-gut cell proliferation monitored by bromodeoxyuridine (BrdU) incorporation—**(A)** T.S. of mid-gut from Cry toxin exposed 24, 48, and 72 h of larvae showing BrdU positive cells. Dextran sulfate sodium injected larvae were used as positive control (PC) and phosphate buffered saline injected larvae were used a negative control (NC). Note the presence of dividing cells with the brown stained nucleus. **(B)** Enlarged portion of mid-gut epithelium from 48 h toxin exposed larvae showing actively dividing cells in the crypt.

The results presented in Figure 3 show few pockets of BrdU-positive cells (brown stained nuclei) were identified in the mid-gut collected from toxin exposed larvae (Figure 3A). The dividing cells which were mostly observed in the basal region of the gut epithelium could be ascribed as mid-gut stem cells (Figure 3B, inset).

### Mid-gut cell death is associated with regeneration

**Figure 4:**
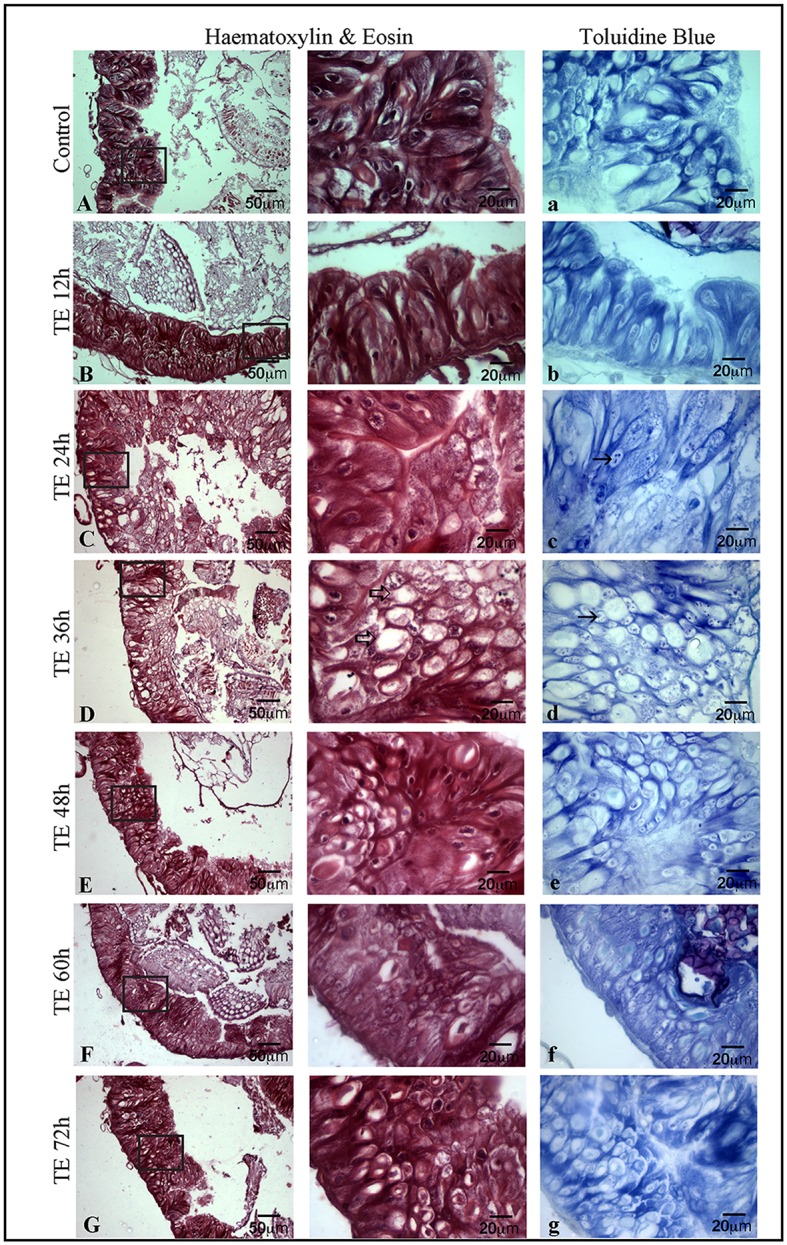
Occurrence of cell death and proliferation in mid-gut demonstrated by haematoxylin-eosin (H & E) and toluidine blue staining, T.S. of mid-gut shows extensive damage in epithelium **(C)** and the presence of a large number of vacuoles (⇒) **(D)** after toxin exposure. T.S. of mid-gut stained with toluidine blue **(A–G)** showing the proliferative cells (→) **(C–D)**. (Scale bars 20, 50 μm).

Figure 4 shows the histological changes seen in toxin exposed larval mid-gut from 12 to 72 h. The mid-gut cells showed degeneration with a condensed nucleus and fairly low cytoplasmic content. In addition, a large number of vacuoles were seen in them. Maximum cell death was observed between 24 and 36 h (Figures 4C,D). However, it was interesting to note that active cell proliferation as depicted by toluidine blue was seen extensively in 36 h Cry toxin exposed larvae (Figures 4C,D). BrdU labeling, histological and toluidine blue staining studies in parallel confirmed that not only cell death but also regeneration occurred in larval mid-gut upon Cry intoxication in *A. janata*.

### Expression of arylphorin in the mid-gut

**Figure 5:**
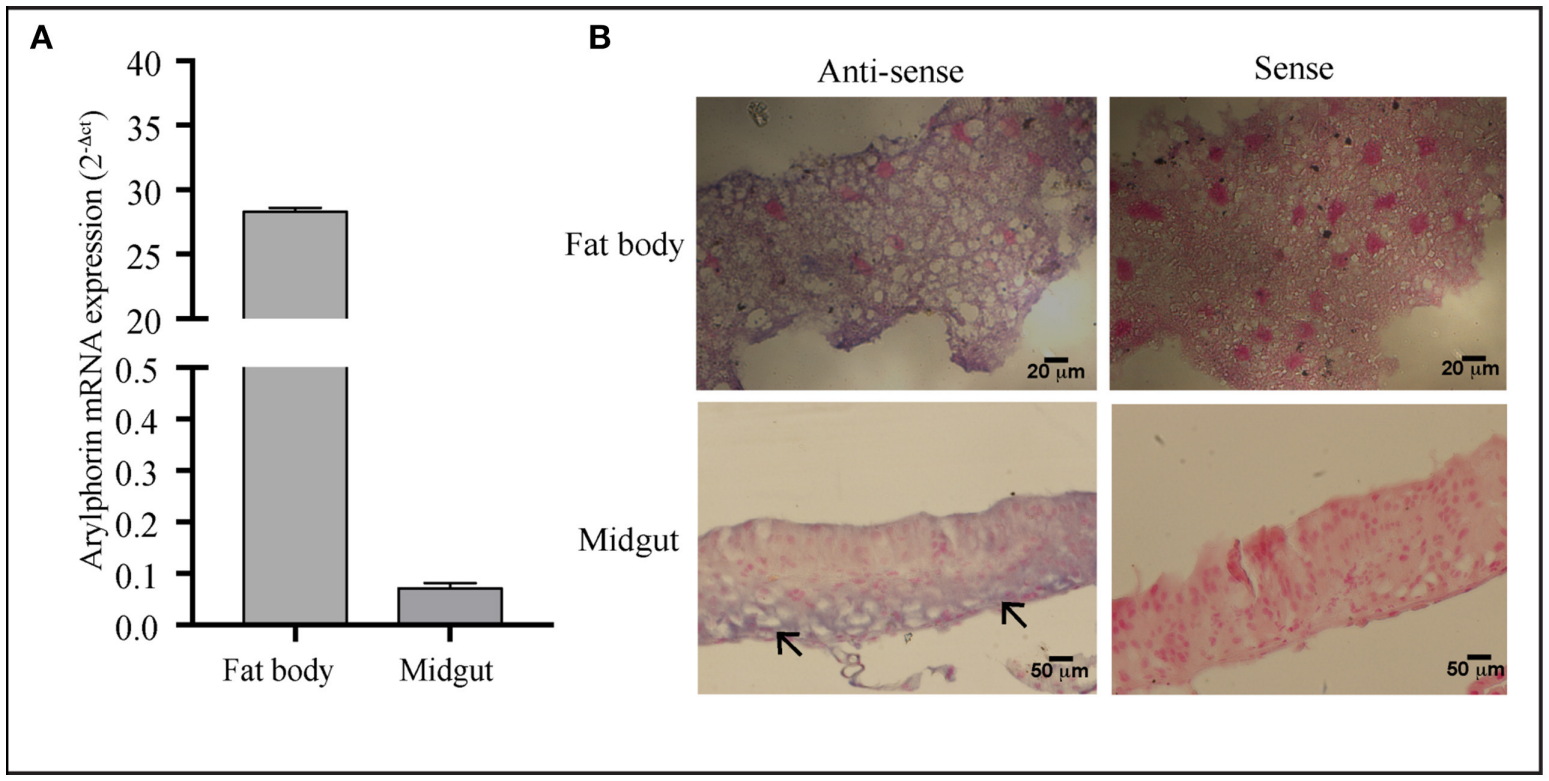
Arylphorin gene expression—**(A)** qPCR analysis showing expression of arylphorin mRNA in the fat body and mid-gut of 3rd instar larvae, **(B)** *In situ* hybridization demonstrated the localization of arylphorin mRNA in the fat body and mid-gut epithelial cells (→) which are primarily observed toward the basal border (Scale bars 20, 50 μm).

Detectable but low expression of arylphorin in the mid-gut as compared to the fat body was observed using qRT-PCR analysis (Figure 5A). In accordance to this, *in situ* hybridization results revealed the localization of arylphorin transcript in the gut epithelium primarily in the basal cells (Figure 5B).

### Cry toxin exposed larval mid-gut displays differential expression of arylphorin

**Figure 6:**
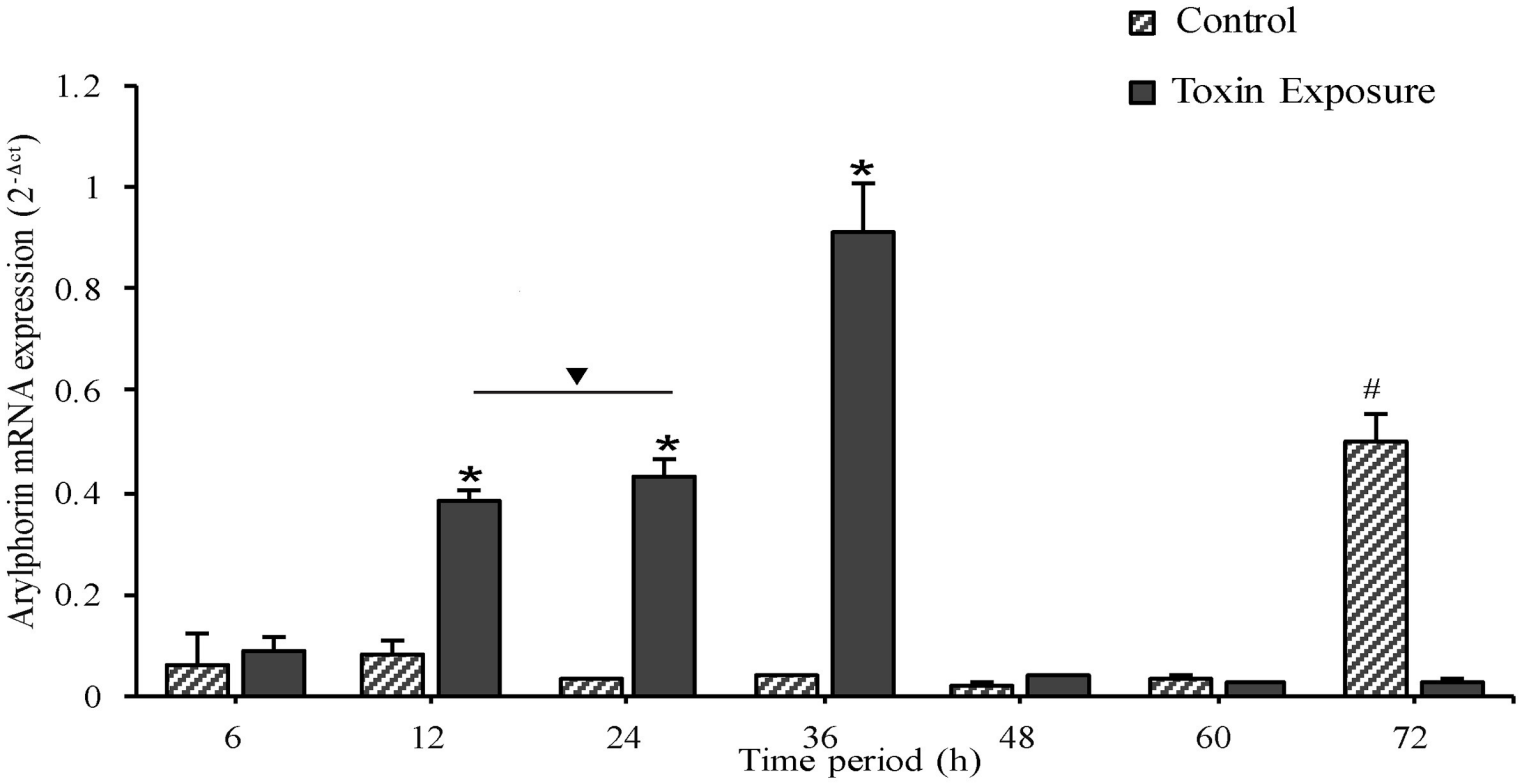
Expression of mid-gut arylphorin mRNA during toxin exposure, A gradual increase was observed from 6 to 36 h of Cry intoxication exposure which declines later to a basal level. Note the higher expression of arylphorin transcript in 72 h control larvae, a period which coincides with molting (from 3rd to 4th larval instar). ^*^p < 0.005 indicates significance between experimental groups, #*p* < 0.005 significant among control groups, ∇ the values are non-significant.

In control insects, arylphorin transcript level was fairly low throughout the time points monitored, but a steep incline was observed at 72 h (Figure 6). On the other hand, arylphorin expression gradually increased from 6 h and reached maximum at 36 h in Cry intoxicated larvae, thereafter the mRNA levels declined significantly in 48 h and remained low till 72 h.

## Discussion

In the present study, the effects of a sub-lethal dose of *Bt* toxin was analyzed through the mid-gut responses in *A. janata* larvae. As observed in this pest and other reported lepidopteran models, *Bt* exposed larvae usually have fairly low digestive activity concomitant with their reduced body weight associated with altered growth and development (Hernández-Martinez et al., 2010). DOR *Bt-*1 toxin administration triggered significant changes in the gut epithelium including nuclear condensation, vacuolization, low cytoplasmic content, and loosening of cell followed by necrosis. These observations corroborated well with an earlier study (Budatha et al., 2007) and suggested that even in *A. janata*, the binding of Cry to mid-gut cell receptors promote cell death. However, there is increasing evidence that Cry toxin induced cell death is a fairly complex cellular response. Zhang et al. (2006) using cabbage looper ovarian cell culture demonstrated that Cry1Ab toxin manifests its effect through Mg^2+^dependent adenylyl cyclase/protein kinase A signaling pathway. Present microscopic and FACS analyses in *A. janata* showed Cry toxin induced necrotic type of cell death in larval mid-gut under *in-vivo* condition without DNA fragmentation (a hallmark of apoptosis). Recently, using toxin-host interaction system in *Caenorhabditis elegans*, Cry6Aa was shown to promote cell death through necrotic signaling pathway (Zhang et al., 2016). In addition, many bacterial toxins such as β-toxin from *Clostridium perfringens*, delta-toxin from *Clostridium septicum*, and VacA from *Helicobacter pylori* were shown to cause cell necrosis (Knapp et al., 2010; Radin et al., 2011; Sridharan and Upton, 2014; Seike et al., 2016). Taken together, the results obtained in the present study confirm that exposure to sub-lethal doses of Cry toxin triggers cell death in larval mid-gut of *A. janata* through necrosis.

Inspite of mid-gut cell death, larva did survive the exposure to sub-lethal doses of *Bt* toxin. Replenishment of injured cells is facilitated by normal feeding leading to recovery and metamorphosis (Spies and Spence, 1985; Loeb et al., 2001), while high doses of *Bt* toxin are known to kill insect pests (Tabashnik et al., 2013). Like many other tissues, renewal of epithelium is carried out by regenerative cells localized in the basal crypts, termed as mid-gut stem cells. These are the only cell present in gut capable of division and thus represent the sole source of new cells during tissue repair and growth. Continuous replacement of damaged cells by regenerative cells under toxin environment is likely to cause an alteration in cellular properties, leading to the development of resistance in insects against Cry formulations (Martinez-Ramirez et al., 1999; Castagnola and Jurat-Fuentes, 2016). Majority of cases of resistance against Cry toxins, reported reduction in binding of *Bt* toxins to their specific mid-gut receptors, i.e., cadherins, aminopeptidases, and alkaline phosphatases, which resulted in cross-resistance to Cry toxins sharing common altered receptor site (Morin et al., 2003; Pigott and Ellar, 2007; Zhang et al., 2009). However, Cry toxin resistance involving alterations in toxin processing by mid-gut secretion was also reported (Oppert et al., 1997; Caccia et al., 2012).

A number of growth factors promoting mid-gut cell culture regeneration have been reported, which include fetal bovine serum, ecdysteroids (Loeb, 2010), mammalian growth-promoting factor (Nishino and Mitsuhashi, 1995), alpha-arylphorin, MDFs (Blackburn et al., 2004; Hakim et al., 2010), and AlbuMAX II (Castagnola et al., 2011). The proliferation and differentiation of mid-gut epithelial cell seen upon Cry intoxication in *A. janata* might have been triggered and modulated by similar factors. Hexamerin proteins, particularly exogenous arylphorin (fat body synthesized arylphorin) were shown to promote proliferation of mid-gut cells in culture (Hakim et al., 2007). Mid-gut transcriptome profile showed up-regulation of arylphorin transcripts in the Xentari™ resistant *Spodoptera exigua* larvae (Hernández-Martinez et al., 2010). The transcriptome analysis with *A. janata* larval mid-gut also revealed up-regulation of arylphorin upon Cry intoxication (Unpublished data). In addition, in the present study *in situ* hybridization provided direct evidence for the localization of arylphorin transcripts in the actively dividing cells of mid-gut, which were primarily confined in the basal region of the epithelium. Expression analysis using Cry intoxicated larva mid-gut further showed time dependent increase in endogenous arylphorin gene expression during active cell proliferation, which was not only seen upon Cry intoxication but also during molting in control larvae where regeneration has been well-documented (Webb and Riddiford, 1988; Kawaguchi et al., 1993).

The present study, not only demonstrated cell death and damage of mid-gut epithelium upon Cry intoxication but also proliferation of stem/regenerative cells that allow the larvae to repair its gut epithelium and generate differentiated cells to facilitate their survival. This is further substantiated by the presence of a large number of BrdU positive cells in the basal crypts of the gut epithelium, while toludine blue staining reveals a sizable increase in the number of proliferative cell upon Cry intoxication in time dependent manner. Given the versatility of arylphorin, its presence and altered expression in the mid-gut during Cry toxin exposure, it is possible that a low concentration of arylphorin produced endogenously in mid-gut is critical and probably inculcates mitogenic effect during the Cry intoxication. Further characterization of the gut regenerative process in the future will help in obtaining a better understanding of the resistance mechanism.

## Author contributions

Conceived and designed the experiments: AD, VC, and ND; Performance of the experiments: VC and ND; Analysis of the data: AD, VC, ND, and RC; Suggestion and help for *in-situ* analysis: BS; Manuscript writing: VC and ND; Manuscript editing: AD, BS, and RC.

### Conflict of interest statement

The authors declare that the research was conducted in the absence of any commercial or financial relationships that could be construed as a potential conflict of interest.
